# Employment of a Milk Diet in Diseases of the Heart

**Published:** 1881-01

**Authors:** 


### THE EMPLOYMENT OF A MILK DIET IN DISEASES OF THE HEART.

AFTER making a classification of diseases of the heart, which it is unnecessary to reproduce here, M. Potain, gives the following as the conclusions at which he has arrived:

Milk diet is especially efficacious in secondary diseases of the heart, hypertrophies or simple dilations, having a renal or gastric origin.

The especial value of milk diet in these cases consists in the fact that the stomach and kidneys are permitted to rest during this treatment. Hence, in order that good results may be attained, the treatment should be continued for some time. Similarly good results may be expected in palpitation or other reflex disturbances of the heart when they are due to gastric derangement. Milk has a diuretic effect, and hence is useful in cases of dropsy from renal disturbance or from secondary inflammation of serous membranes.

Finally, milk may be exceedingly useful, because it is better tolerated than other nourishment, and because it is sufficient to sustain life.—*Virginia Medical Monthly.*

